# The Effect of Local Orientation Change on the Detection of Contours Defined by Constant Curvature: Psychophysics and Image Statistics

**DOI:** 10.3389/fpsyg.2016.02069

**Published:** 2017-01-17

**Authors:** Sieu K. Khuu, Joey Cham, Anthony Hayes

**Affiliations:** ^1^School of Optometry and Vision Science, University of New South WalesSydney, NSW, Australia; ^2^Department of Psychology, The University of Hong KongHong Kong, Hong Kong

**Keywords:** contour integration, curved contour detection, spatial vision, visual psychophysics, image statistics

## Abstract

In the present study, we investigated the detection of contours defined by constant curvature and the statistics of curved contours in natural scenes. In Experiment 1, we examined the degree to which human sensitivity to contours is affected by changing the curvature angle and disrupting contour curvature continuity by varying the orientation of end elements. We find that (1) changing the angle of contour curvature decreased detection performance, while (2) end elements oriented in the direction (i.e., clockwise) of curvature facilitated contour detection regardless of the curvature angle of the contour. In Experiment 2 we further established that the relative effect of end—element orientation on contour detection was not only dependent on their orientation (collinear or cocircular), but also their spatial separation from the contour, and whether the contour shape was curved or not (i.e., C-shaped or S-shaped). Increasing the spatial separation of end-elements reduced contour detection performance regardless of their orientation or the contour shape. However, at small separations, cocircular end-elements facilitated the detection of C-shaped contours, but not S-shaped contours. The opposite result was observed for collinear end-elements, which improved the detection of S- shaped, but not C-shaped contours. These dissociative results confirmed that the visual system specifically codes contour curvature, but the association of contour elements occurs locally. Finally, we undertook an analysis of natural images that mapped contours with a constant angular change and determined the frequency of occurrence of end elements with different orientations. Analogous to our behavioral data, this image analysis revealed that the mapped end elements of constantly curved contours are likely to be oriented clockwise to the angle of curvature. Our findings indicate that the visual system is selectively sensitive to contours defined by constant curvature and that this might reflect the properties of curved contours in natural images.

## Introduction

In visual scenes, contours are a collection of spatially linked line elements that are associated based on a small number of common properties such as regular continuity, orientation, depth, closure, and proximity (see e.g., Marr, 1982; Field et al., 1993; Kovacs and Julesz, 1993; Khuu et al., 2015, 2016). Spatial contours are salient attributes of the visual scene as they may signify spatial layout, which contributes to defining the boundaries of objects and perceptual organization (Marr, 1982). Particularly, contour structure informs the observer about the shape, position, and form of an object and serves as one of the most valuable cues used to segment an object from its background.

To date, a good deal of psychophysical, physiological, and neural-imaging research has characterized the neural processes underlying contour integration and analysis. The classic study of Field et al. (1993) demonstrated that the visual system's ability to detect contours is highly dependent on associative principles related to the relative orientation, length, and element separation, of contour elements. This work introduced the concept of a contour association-field to model the optimal relationship between local edge-elements in which contour grouping occurs. Particularly, this model holds that the integration of contours is underpinned by the principle of “good-continuation,” such that a train of local pairwise contour elements are likely to be grouped to form a contour if they are placed in close proximity to each other, have the same (or similar orientation tuned to a particular range), polarity, and phase; pairwise edge elements that largely differ in these properties are not grouped. In addition to good-continuation, it has been shown that the perception of contours is further enhanced if they are closed. For example, Kovacs and Julesz (1993) reported a detection advantage to closed contour stimuli compared to those with open structures (see also Mathes and Fahle, 2007; c.f., Tversky et al., 2004). This preference to closed contour might reflect the fact that real objects and salient features in the visual world (such as fruits and faces), which tend to have complete edge structures.

Single-cell recording studies have indicated that the physiological basis of the association-field might lie in the short- and long-range connectivity of local edge sensitive neurons in the primary visual cortex (Bosking et al., 1997; see Hess and Field, 1999 for a review). Additionally, recent EEG-fMRI and MEG work (see Tanskanen et al., 2008; Mijovic et al., 2013) have confirmed the importance of early visual areas in the perception of contours, but also highlight the involvement of feedback from higher cortical areas such as the lateral occipital complex. Previous studies have shown that the processing of spatial contours (and perhaps the development of the abovementioned neural connections which might underpin contour grouping principles) is not optimal at birth, but rather follows a slow developmental perceptual process over a number of years (e.g., Kovacs et al., 1999; Gervan et al., 2011; Taylor et al., 2014). Interestingly, the contribution of closure and good-continuation to the detection of contours are dissociable and appear to be follow different stages of development (see Gerhardstein et al., 2004; Hipp et al., 2014).

Recent work has sought to draw a relationship between the computational rules and the neural implementation of contour integration with the statistics of contour structures in images of the natural environment. Some insight into the possibility that contour perception is optimally tuned to the natural environment have been provided to date, though only by a handful of studies (e.g., Parent and Zucker, 1989; Geisler et al., 2001; Elder and Goldberg, 2002). For example Geisler, et al. investigated the co-occurrence probabilities of all geometrical relationships between edge-pairs in natural images regardless of scale. They noted that collinear pairings (i.e., straight lines) were most prevalent (at short distances) and the orientation statistics conformed well to the association-field model of Field et al. (1993). Additionally, Geisler et al noted that pairwise associations between elements separated by moderate distances were not collinear, but cocircular (i.e., edge pairs are tangent to a common circle). This finding agrees with the conclusions of Sigman et al. (2001) who confirmed that the relationship between relative orientation and relative position of two adjacent line elements is also evidently cocircular.

**Figure 1:**
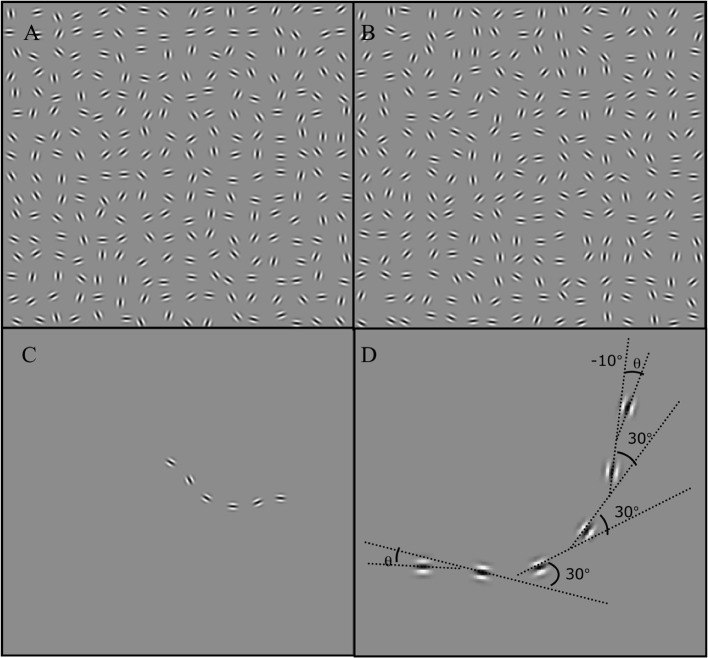
**In (A)** shows a target-present interval. In **(B)** shows a target-absent interval where only randomly oriented Gabors are present. In **(C)** the target contour that is present in **(A)** is shown in the absence of noise Gabors. **(D)** Configuration of a representative target contour. This contour has a curvature angle of 30° with the direction of end elements, given by θ, set to −10°.

While the abovementioned image analyses have made important contributions to the processing of curved contours they are limited to pairwise associations (i.e., between two elements that are either adjacent or non-adjacent and separated over a larger distance) and do not immediately reveal the statistics of longer contours comprising of many elements that conform to extended forms such as constant curvature. Contours that are constantly curved are trains of elements in which elements are equally displaced with their orientations following a constant angular path (see Figure 1A) and thus, the relationship between pairwise elements is cocircular. If the element difference angle is 0°, the contour is a straight line; the extent of curvature is indicated by the magnitude of the angular difference between adjacent elements within the train. Previous research (e.g., Kovacs and Julesz, 1993; Pettet, 1999; Mathes and Fahle, 2007) has indicated that such curved contours are abundant in natural scenes and are more likely to be correlated with the edges of objects that are meaningful to the visual system such as faces and fruit, or the corners or junctions of objects. The curvature that characterizes such objects might signal a meaningful unit that perhaps conforms to the Gestalt principles of “continuity” and “smoothness” and might contribute to “closure”—a tendency to perceive a whole stimulus from incomplete parts. Indeed, previous studies have shown that the visual system is preferentially sensitive to the curvature characteristics of contours (e.g., Pettet, 1999; Khuu et al., 2016). For example, Pettet (1999) showed that path smoothness and the angle of curvature were important in the perception of curved contours as they directly facilitate contour detection and sensitivity. Elder and Goldberg (2002) argued that preferential coding of extended curved contour structure might provide a means of grouping elements over large distances which is advantageous and overcoming local variations in luminance and occlusion which might otherwise disrupt contour detection (see also Geisler and Perry, 2009).

Previous studies have attempted to account for the preferential processing of curved contours by proposing a change in the neural connectivity of edge-selective units in the visual cortex. As mentioned, Bosking et al. (1997) revealed that orientation tuned cells in V1 are associated through short and long range lateral fibers, to form a network capable of extracting contour information. In the case of contours with constant curvature, it is possible that such lateral connections between orientation cells act to code contours through the propagation of curvature structure by changing the weighting of their local connections (e.g., Yen and Finkel, 1998). Particularly, to ensure continuity and smoothness, the association field of orientation detectors might be re-modified so that they are biased in the angle of curvature, and thereby, provide preferential sensitivity to such contours. Similarly, Pettet et al. (1998) argued that curvature structure is coded through the facilitation of local elements that are in close proximity and similarly oriented, and in which reverberating activity along in this circuitry produces an enhancement in the detection of curvature structure. They demonstrated that a computational model that considered curvature, separation and curvature change as facilitating factors in the grouping of elements provided a good estimate of human contour detection performance. Thus, these aforementioned models consider contour integration beyond pairwise associations (i.e., position and relative orientation) of oriented-elements, and proposed that grouping is influenced by more extended rules such as contour smoothness, continuity and perhaps closure.

In the present study, our goal was to further clarify whether the visual system is sensitive contours defined by constant curvature and draw inferences about the statistics of these contours in natural images. The importance of this work is that its twin approach allows for the direct establishment of the relationship between human sensitivity and the statistics of contour structure in natural images, and thereby provides evidence that the visual system has developed mechanisms to encode the statistics of visual-image patterns that it usually encounters.

The possibility that preferential sensitivity to curved contours arises because of a bias in the association field in the curvature direction leads to a number of predictions about their detection. If such an operation accounts for the detection of curved contours, an expectation is that because the curvature structure propagates along the extent of the contour, then end-elements tilted or aligned with the curvature angle (and not collinear with the previous contour element) will facilitate detection. However, deviation from the curvature angle will systematically reduce contour sensitivity, as contour end elements are less likely to be grouped. In Experiment 1 we tested this prediction by using the methods of Field et al. (1993) to examine the ability of observers to detect curved contours as a function of the contour curvature angle and the orientation of end elements. In Experiment 2 we further investigated the degree to which the orientation of end elements facilitated contour detection by examining how contour detectability changed as a function of their spatial separation from the contour. Previous studies have indicated that increasing the inter-element distance reduces contour detectability (see Field et al., 1993), and in the present study, we sought to specifically examine this effect in the context of curved contour processing by comparing performance between contours that are constantly curved (C-shaped) and those that are not (S-shaped). These two contour types were examined as it was expected that their detection might be dependent on the relative orientation of end elements. Finally, to provide initial insight into the possible relation between contour detection and their statistics in natural images, we performed a simple image analysis, which for the first time mapped extended contours of different constant curvatures and the orientation frequency of end elements to establish the “continuity” of curvature structure in natural scenes.

## Experiment 1: the effect of end element orientation and contour angle on curved contour detection

In Experiment 1 we examined whether the detectability of contours with different degrees of curvature is affected by the local orientation of end elements. Stimuli were trains of six Gabors elements with the central four elements following a constant angular change which resulted in a smooth curved path (see Figure 1). In Figure 1, curved-contour structure is evidently conveyed, but the contour was not closed. Contour closure has been shown to facilitate contour detection (Kovacs and Julesz, 1993; Mathes and Fahle, 2007), and this advantage was minimized by using open and short contour-fragments to directly investigate the specificity of curved contour processing. As mentioned, contour sensitivity was measured in terms of the ability of the observer to detect a contour placed within a field of randomly oriented Gabors, and this process was repeated as a function of the magnitude of the curvature angle and the orientation of end elements. We predicted that if the visual system specifically codes curved contour structure, contours with end elements oriented in the curvature direction will be more detectable than tilted in another direction. However, if contour detection operated in a pairwise fashion, then collinear placements of end elements will lead to greater contour sensitivity. These testing procedures offer direct way of assessing the specificity of curved contour processing (beyond simply characterizing their detectability as a function of contour angle, see Pettet, 1999; Beaudot and Mullen, 2003; Khuu et al., 2016), as it directly assesses the propagation of curvature structure along the extent of the contour to affect the coding of elements at its ends.

### Methods

#### Observers

Six observers, with normal or corrected-to-normal vision, participated in this experiment. Except for JC, the other observers were naïve to the purpose of the experiment. The age range of the observers was 22–28. All observers gave their prior written consent (with ethics approval given by the human research ethics committee at the University of Hong Kong) and the study followed the tenets of the Declaration of Helsinki.

### Stimuli

Stimuli were a square field (8° by 8° of visual angle) consisting of 256 oriented Gabor micro-patterns placed on a gray background at a luminance of 45 cd/m2 (see Figure 1A) Each Gabor micro-pattern was given by the product of a circular Gaussian and an oriented sinusoid:

$$\begin{array}{l} G(x,y)\;=\;\exp\;(-\frac{x^{\prime 2}+\gamma^{2}y^{\prime 2}}{2\sigma^{2}})*\cos(2\pi \frac{x^{\prime }}{\lambda }+\psi )\\ where\\ \;x^{\prime }\;=\;x\cos\theta +y\sin\theta \\ \;y^{\prime }\;=\;-x\sin\theta +y\cos\theta \end{array}$$

θ is the orientation, ψ is the relative phase of the element, γ is the spatial aspect ratio, and λ is the spatial frequency of the modulating sinusoid was set to eight pixels (0.125°). The size of the element, is defined by σ, was fixed at four pixels (σ = 4), thus the element at full width and at half-height was approximately 0.125° of visual angle. With the viewing distance of 90 cm, these parameters resulted in elements with a peak spatial frequency of eight cycle/° and a bandwidth of approximately 1.2 octaves (σ = 1/16°).

As mentioned, each target contour consisted of six Gabor elements (see Figure 1D). We chose this length because it provides an adequate approximation of a curved contour (see Figure 1) and prevents the likelihood of the contour forming a circle or wrapping around at high curvature angles. A pilot study established that detection performance remained unchanged for contours equal to and greater than six elements. The four Gabors forming the middle elements of the contour were oriented along one of five contour angles of 0, 10, 20, 30, and 40°. Gabors at the two ends were placed at the same inter-element distance away from the contour, but depending on the condition were located in directions of −30, −20, −10, 0, 10, 20, 30, 40, 50, 60° relative to the orientation of the last element of the contour. Negative values indicated directions away/anti-clockwise, while positive values indicate the direction/clockwise to the angle of curvature. The orientation of the two end elements was fixed and in the same as its direction. The inter-element distance was fixed to 0.5°For contours with curvatures of 10, 20° and 30°, and 0.75°For a 0°Contour. A longer distance is used for a 0°Contour since smaller separations result in a pop-out effect which results in the contour being clearly segregated from the noise background. Increasing the inter-element distance removed this segmentation effect. Figures 1C,D are examples of the target contour stimulus; this contour is set with a contour angle of 30° and with end elements (θ) placed in a direction of −10°.

Similar to the methodology of Field et al. (1993), to ensure that the presence of the contour was not revealed by any possible difference between the inter-element distance between the contour and background noise elements, the element density was carefully controlled by restricting the position of elements to cells of a grid matrix (16 × 16 grid squares). Here an element could occupy any position within a grid square (0.5 × 0.5°), but was restricted such that their centroid must fall within this region. The spatial position of the contour in the grid matrix was randomly determined by repeatedly positing and rotating the contour to search for a location where each contour element fitted within a grid square. Subsequently, each of the empty grid squares was filled with a randomly oriented and positioned element. Because all the elements were distributed over the 256 grid-squares approximately equal local density in the stimulus was maintained. Note that our method of measuring contour detection differs from other procedures that have instead varied the inter element distance of background and or contour elements (e.g., Kovacs and Julesz, 1993; Braun, 1999). While different methods are available, previous studies have clearly demonstrated that all are equally valid in providing a measure of contour detection performance. Our motivation for using the approach of Field et al. (1993) is for want of control of the inter-element distance to ensure that edge elements only differ in their spatial orientation. This was important to the goals of the present study, which primarily sought to investigate the orientation relationship between contour elements in the processing of contour curvature.

Contour stimuli were generated using custom written software in MATLAB (version 7.2) on a 2.5 GHz Macintosh Power PC G5. Stimuli were subsequently displayed on a linearised Mitsubishi Diamond Pro 2070 monitor. Observers viewed the images binocularly at a distance of 90 cm under dim illumination.

#### Procedure

A temporal two interval-forced-choice (2IFC) design was used to examine the detectability of curved contours. In each trial, observers were sequentially shown two stimuli in separate intervals. The order of these stimuli was randomized from trial to trial. One interval contained an image with the target contour in a field of randomly oriented Gabor elements (e.g., Figure 1A). While in the other interval, the stimulus contained only randomly oriented Gabor elements (Figure 1B). Each presentation lasted for 1 s, and they were separated by a 1-s blank period set to the background luminance. The task of the observer was to identify the interval containing the contour and they indicated their response by pressing one of two keyboard buttons. No feedback was given to indicate the correctness of the response. As mentioned, we examined the ability of observers to detect five different contour curvature angles, and each had 10 different end-element orientations. Thus, there were 50 different stimulus configurations. In a block of trials observers were presented with each stimulus configuration 25 times in randomized order (thus in one block there were 1250 trials). Observers completed eight blocks of trials and the results averaged over blocks for each stimulus configuration.

### Results and discussion

**Figure 2:**
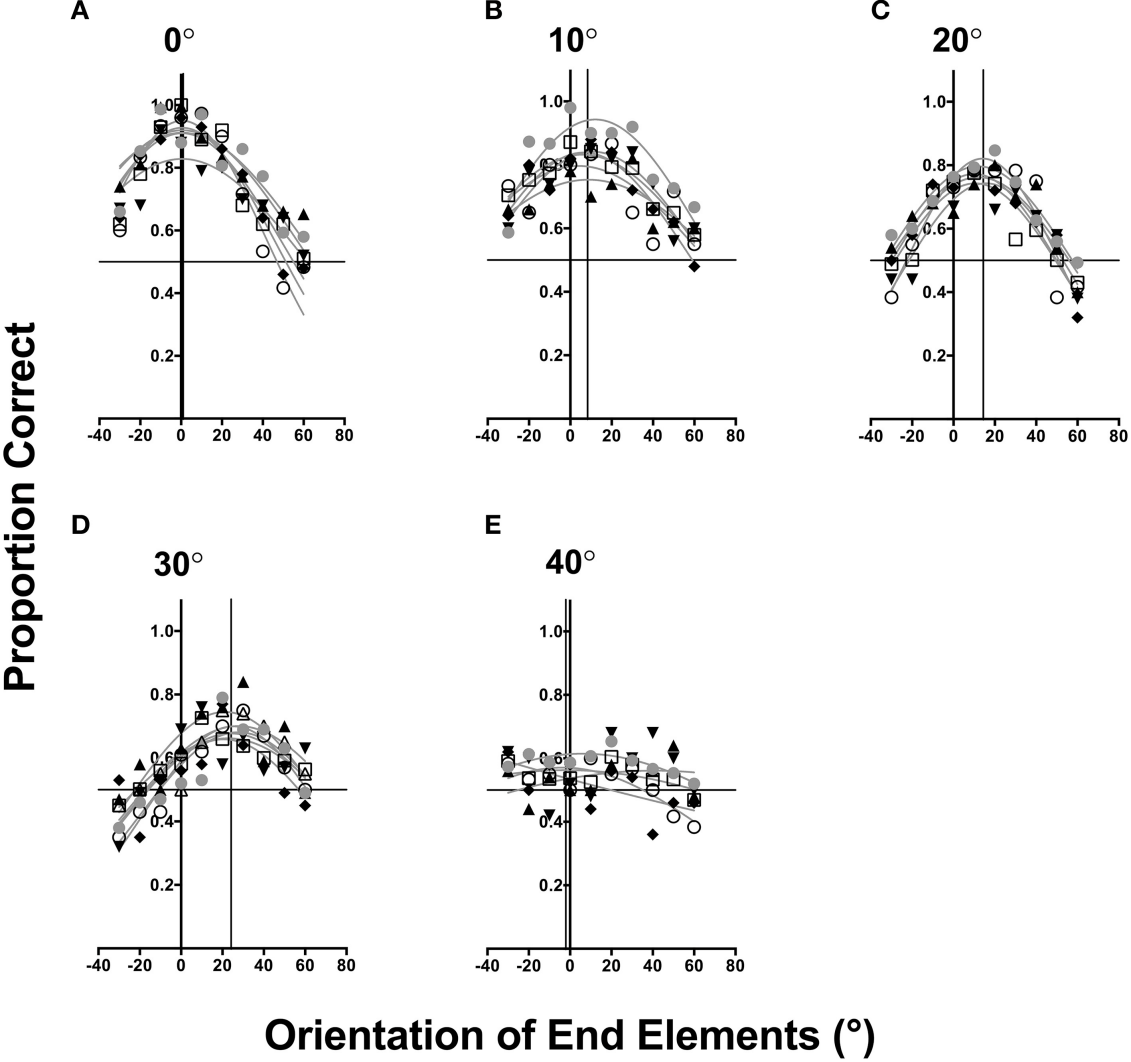
**Detection sensitivity to different curved contours (A–E)** plotted as a function of the orientation of end elements. Data from different observers are represented by different symbols. For each observer a Gaussian function was fitted. Vertical black lines represent the observer average Gaussian fit mean, while solid horizontal lines represent chance performance.

Figure 2 plots the data of the six observers (different symbols). Different panels show data for different contour angle conditions (Figures 2A–E). For each contour angle, the proportion of times in which the interval containing the contour was correctly identified is plotted as a function of the end-element orientation. The pattern of results for all observers, while demonstrating a degree of inter-observer variability, was similar: the ability to detect contours is dependent on the orientation of end elements with performance tuned for a range of values. A two-way repeated measures ANOVA was conducted (after the data were converted to continuous by using a logit transform) to examine the effect of changing the contour angle and orientation of end elements on detection performance. This analysis reported a main effect of both the contour angle [*F*_(4, 300)_ = 110.86, *p* < 0.0001] and end-element orientation [*F*_(9, 300)_ = 42.44, *p* < 0.0001]. However, a significant interaction effect was also observed [*F*_(36, 300)_ = 7.18, *p* < 0.0001], which indicated that changing the orientation of end elements on contour detectability was dependent on the contour angle.

To provide an indication of the relationship between contour detectability and end-element orientation, a Gaussian function of the form was fitted to the data for each observer (black solid lines):

$$\begin{array}{l} Y=Ae^{-0.5\frac{{\left(X-M\right)}^{2}}{SD}} \end{array}$$

where A was the amplitude of the distribution, *M*: the mean, and *SD*: the standard deviation of the distribution; these parameters provided a measure of the overall ability of the visual system to detect contours, the orientation of end elements leading to best detection, and the range of end-element orientations likely to facilitate contour detection, respectively.

A general observation of Figure 2 is that as the contour angle increases, there is an overall reduction in the ability to detect contours (indicated by a reduction in the observer average A (error is indicative of 1 standard error of the mean) of the Gaussian fits: 0°: *A* = 0.908 ± 0.017; 10° *A* = 0.834 ± 0.063; 20°: *A* = 0.773 ± 0.031; 30°: *A* = 0.687 ± 0.032; 40°: *A* = 0.566 ± 0.033), but no systematic change in the range of end-element orientations facilitating detection (with observer average SD of the Gaussian distribution approximately 61°). A one-way ANOVA indicated that increasing contour angle significantly decreased contour detection performance [*F*_(4, 25)_ = 61.76, *p* < 0.0001]. For a contour angle of 40° (Figure 2E), the detection performance decreases to near chance level and is not dependent on the orientation of end-elements given by a large observer average *SD* of 92° and the mean of the distribution is 2.76° ± 9.39. This finding indicates that observers were unable to detect contours of high curvature under the stimulus conditions employed by the present study. Note that this result is different from Pettet (1999), who showed that contours of 40° were detectable with proportion correct detection at approximately 0.8. An explanation for this difference between the present study and those of Pettet stems of the task used to measure contour detection. Pettet used a method in which the position of the target contours was always fixed within a small region of noise, and in a spatial two alternative forced choice observers judged which region, either to the left or right of fixation, contained the contour. Since the position of contour was easily discernable (as spatial uncertainty was minimized) using this method, observers might have performed this task with relative ease. However, in our task, which adheres to the methods of Field et al. (1993), the position of the to-be-detected contour was randomly determined from trial to trial in a large field of noise. Thus, this task is much harder since observers had to first localize the contour, before discerning its contour structure. Our findings are consistent with the work of Beaudot and Mullen (2003), who observed that the detection of contours with different curvatures systematically decreased with the contour angle as well as element separation.

In Figure 2A, for contours with a curvature of 0°, the observer average orientation of end elements leading to the best performance (M, given by the solid vertical line) is approximately 0° ± 0.051 SEM and is the same as the angle of curvature. However, the ability to detect contours systematically decreases as a function of the end-element orientation, and for angles deviating >60° performance is approaching chance (horizontal dashed-line) for some observers.

The pattern of results for larger contour angles (as shown in Figures 2B–D), is one in which the orientation of end elements leading to best detection performance is clockwise to the angle of curvature. For a contour angle of 10°, the average orientation of end elements resulting in maximum detectability was approximately 8.054° ± 1.277 SEM, while for contour angles of 20 and 30°, maximum detection was noted for end-element orientations of approximately 13.63° ± 0.886 SEM and 23.618° ± 1.099 SEM respectively (also see **Figure 6F**). Note however that the orientation of end elements resulting in the greatest detectability was slightly less than the contour angle. These results suggests a detection bias toward curved contours: end elements that are oriented in the contour angle direction are more detectable relative to the opposite direction (for the same level of orientation change, such as −20 vs. 20°), and this finding is in agreement with previous work (e.g., Pettet et al., 1998; Mathes and Fahle, 2007) showing that fewer changes of direction along a contour (maintaining smoothness) produces better detection performance. In addition, weaker detection performance is observed with end-element orientations that were large or in the opposite direction to curvature angle. An explanation for this is that the visual system is sensitive to the contour angle and end-elements displaced anti-clockwise from the direction of the angle of curvature are not grouped/integrated to detect the contour (see General Discussion).

The findings of Experiment 1, revealed two noteworthy findings. First, contours of less curvature, those that have angles of <40° are most detectable. Second, sensitivity to contours is dependent on the continuity and smoothness of the contour. If the orientation of end elements is systematically changed relative to the contour angle, sensitivity is affected such that clockwise orientation relative to the curvature direction leads to greater detectability.

## Experiment 2: the effect of inter-element-distance on the detection of curved and open contours

In Experiment 1, we demonstrated a detection advantage for curved contours with end elements oriented clockwise to the contour angle. In Experiment 2, we sought to further investigate this effect by determining the spatial limits over which elements are associated with the contour to facilitate its detection. Previous studies have well established that increasing the spatial distance between all elements reduces contour detection (see Beaudot and Mullen, 2003). Here, we adopt a modification of this approach by only changing the spatial distance of end elements from the curved contour. Importantly, this allowed us to directly establish the association range over which end elements are grouped and the propagation extent of curved contour structure.

**Figure 3:**
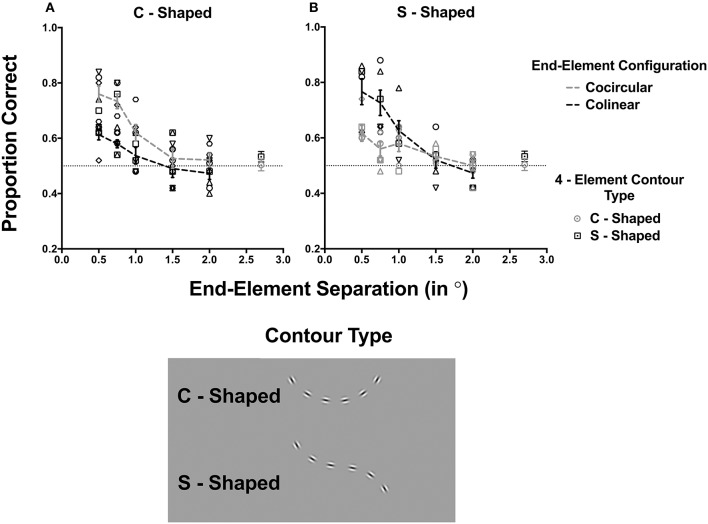
**The proportion of times observers correctly identified the contour plotted as a function of the end-element separation and separately for C-shaped and S-shaped contours**. Each symbol represents the results for one observer. The conditions in which end elements were collinear and cocircular to the contour are represented by black and gray colored symbols respectively; the observer average for these conditions are given by the dashed lines. Error bars signify 1 standard error of the mean. The dotted horizontal at 0.5 proportion correct represents chance performance.

We examined and compared the effect of increasing the inter-element distance of end elements for two contour configurations in which the contour followed a constant angular change producing a “C-shaped” curve as in Experiment 1, or changed in the sign of curvature at the midpoint of the curve which produces an “S-shaped” contour configuration (See the legend of Figure 3). We reasoned that whether end elements were oriented in the contour angle or collinear with the previous element of the contour might differently affect the detection of these two contour types. Particularly for C-shaped contours end elements oriented in the contour angle direction will facilitate contour detection, but a collinear relationship with the previous element of the contour will reduce detection performance. These outcomes were previously shown in Experiment 1. On the other hand, for S-shaped contours, because local elements placements do not follow a constantly curved path, we predict the opposite outcome. Here collinear end elements might facilitate the detection, and end elements deviating from a collinear relationship will reduce performance. Note that the pairwise relationship (either collinear or collinear) between end elements and the last elements of the contour is the same regardless of whether the contour is C-shaped or S-shaped. Thus, any differences in performance between these two contour types can be attributed to the shape of the contour and whether the visual system is specifically sensitive to curved contours.

### Stimulus and procedures

#### Observers

Six observers with normal or corrected to normal visual acuity participated in the present study. They were different observers to those who participated in Experiment 1. All were experienced observers, but naïve to the purposes of the study.

Stimuli used in Experiment 2 were similar to those used previously, but we examined only contours with an angle of 30°. In separate conditions, end elements were either collinear (i.e., 0°) with the last element of contour or cocircular (30°). For both C-shaped and S-shaped contours, the spatial position of the end element relative to the contour was systematically varied from 0.5, 0.75, 1, 1.5, and 2°, and contour detection was measured using the methods and procedures of Experiment 1. Particularly, observers were required to detect contours placed within a random noise field and observers repeated this 50 times for each condition. As there were five separations, two contour types, and two end element orientations, there were 20 different stimulus conditions. Observers performed these conditions in a randomized order broken up over two 1-h experimental sessions. In addition to these conditions, we measured the detection of C-shaped and S-shaped contours without end elements, and therefore they only comprised of the four central elements. These conditions served as a baseline that allowed us to determine the degree to which the addition of end elements increased or decreased contour detection performance.

### Results and discussion

The proportion of times in which observers were able correctly to detect the contour is plotted in Figure 3 for different element separations and for C-shaped and S-shaped contours. In each panel, conditions in which end elements were cocircular or collinear are indicated by gray and black symbols respectively. Data for different observers (different symbols) are shown as well as the mean data, which is represented by the gray (cocircular), and black (collinear) dashed lines. Error bars signify 1 standard error of the mean. Data for the two baseline conditions in which there were only four contour elements that were C-shaped or S-shaped are shown as open circles and squares respectively. The left and right panels (Figures 3A,B) present the results for C-shaped and S-shaped contours respectively.

A three-way repeated measures ANOVA was initially performed to determine the overall effect of contour shape (C- or S-shaped), end-element orientation (0 or 30°) and separation (0.5, 0.75, 1, 1.5, and 2°) on contour detection performance. Importantly, this analysis observed a significant interaction effect of end-element orientation, shape and separation [*F*_(4, 100)_ = 7.6, *p* < 0.0001]. This confirmed that effect of changing the end-element separation on the detection of differently oriented end-element contours was dependent on its shape. Evident in Figure 3 increasing the separation of end-elements decreased contour detection performance, but the degree to which appears to be dependent on the orientation of end elements, but this data trend is opposite for the two contour shapes.

As there was no effect of contour shape [*F*_(1, 100)_ = 0.51, *p* = 0.822], and because of the contrasting effect between the two contour shapes, we undertook two separate two-way repeated measures ANOVA for the two different contour types (C-shaped vs. S-shaped) conditions to determine the differential effect of end-element orientation (collinear vs. cocircular) and separation (0.5–2°) on their detection. For both contour types, these analyses revealed a main effect of end-element orientation [C-shaped: *F*_(1, 25)_ = 14.37, *p* = 0.0008; S-shaped: *F*_(1, 25)_ = 9.99, *p* = 0.0041] and end-element separation [C-shaped: *F*_(4, 25)_ = 11.37, *p* < 0.0001; C-shaped: *F*_(4, 25)_ = 15.98, *p* < 0.0001]. However, there was a significant interaction effect for both contour types [C-shaped: *F*_(4, 25)_ = 3.41, *p* = 0.0235; C-shaped: *F*_(4, 25)_ = 3.8, *p* = 0.0139], which indicated that the effect of changing the end-element separation was dependent on the whether the end-element orientation was collinear or cocircular. Note in Figures 3A,B, changing the element separation had a greater effect on one of the two end-element orientation conditions.

A number of findings are evident in Figure 3. When the contours comprised of only the 4 central elements configured so that they were either C-shaped (open circle) or S-Shaped (open circles) performance was close to chance level (dotted horizontal line). Contour detection improved when elements were added to the ends of the contour for both C-shaped and S-shaped contours, but detection performance decreased with increasing separation and reaches chance levels at the largest separations. Here, detection performance is similar to the 4-element baseline conditions. This suggests that at large separations end elements no longer contribute to its detection.

At small separations, detection performance was additionally dependent whether end elements were collinear or cocircular in orientation. When the contour was C-shaped (Figure 3A) end elements oriented clockwise (i.e., cocircular) to the contour angle, contour detection was superior to when they were collinear. Sidak's *post-hoc* comparisons test (corrected for multiple comparisons, alpha = 0.05), which compared the detection performance between cocircular and collinear contour conditions (at each end-element separation), revealed a significant difference between the two end-element orientation conditions for separations equal to and <1° (0.5°: mean difference 0.14, *p* = 0.0219; 1°: mean difference 0.19, *p* = 0.0017). However, when the contour was S-shaped (Figure 3B), the opposite pattern of results was observed. Here contour detection with collinear end-elements was superior to when end elements were cocircular. Here Sidak's *post-hoc* comparisons test showed a significant difference between the two end element orientation conditions for separations of <1° (0.5°: mean difference −0.15, *p* = 0.0152; 1°: mean difference −0.17 *p* = 0.0061). In conclusion, the dissociable effect of end-element orientation on the detection of C-shaped and S-shaped contours suggests that the visual system is sensitive to the constant curvature structure of the contour and argues against strict pairwise associations between adjacent contour elements.

### Analysis of curved contours in natural images

As mentioned in the introduction, previous studies have sought to understand human detection of contours by clarifying the relationships between edges in statistics of natural images. Particularly Geisler et al. (2001) and Sigman et al. (2001) noted that pairwise edge co-occurrence probabilities tend to be collinear as well as cocircular. While making an important contribution to this area of research, the studies by Geisler et al. (2001) and Sigman et al. (2001) are restricted to a statistical analysis of pair-wise association between oriented features. This limitation gives these analyses power since the assumptions about the nature of significant image structure are minimized. However, this type of pair-wise analysis cannot immediately reveal the statistics of more extended contour structures, notably extended contours that follow some constantly curved path, much like the contours investigated in the present study. However, Geisler et al. (2001) noted that occurrence frequencies across a number of edge element separations followed a smooth path of curvature suggesting that extended contours in natural scenes are likely to be smooth. Indeed, Lawlor and Zucker (2013) have recently shown that curvature structure might be a characteristic of the co-occurrence probability of contours defined by three edge elements.

In the present study, we undertook preliminary steps to specifically and only characterize the statistics of contours that follow a constant angle of curvature in natural images. This analysis is not intended to be exhaustive but represents an initial characterization of the statistics of curved contours in natural images. As a first step we conducted a simple analysis (using the procedures of Geisler et al., 2001) of the geometrical relationship of line elements in a set of natural images with the goal of mapping contours of different curvatures, and revealing the orientation frequency of line elements that are adjacent to the ends of mapped contours. Our psychophysical data indicated that contours with end elements oriented clockwise to the angle of curvature are more detectable, and our specific goal was to determine whether this characteristic of human contour perception might be reflected in the orientation statistics of end elements in natural images.

#### Picture preparation

**Figure 4:**
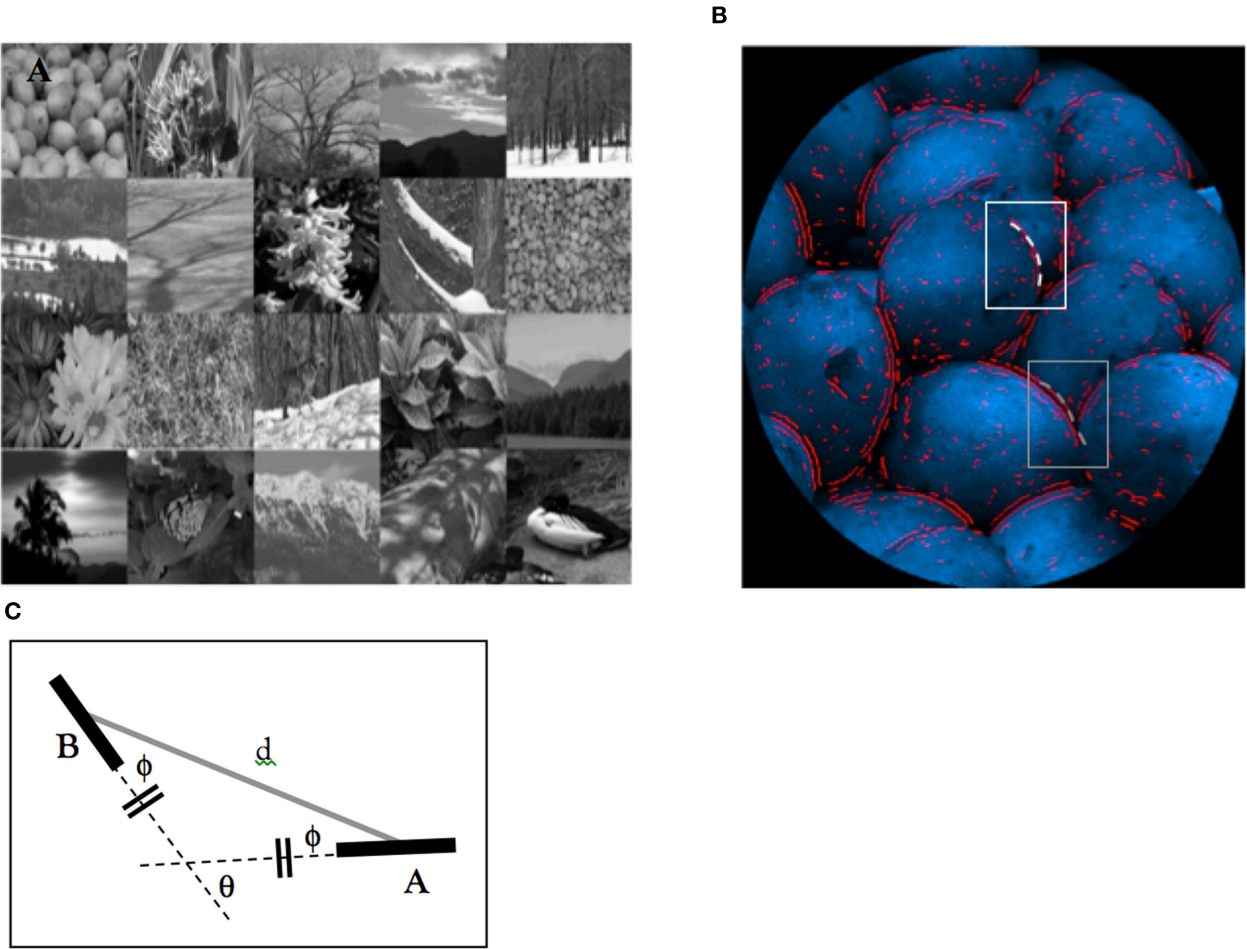
**Image preparation. (A)** Examples, of images of natural scenes. **(B)** Red lines show edge-elements extracted. In **(B)**, white and gray-boxed areas represent a mapped contour that is continuous (and marked by our search algorithm) and one that is not continuous. **(C)** A schematic representation of a “continuous” edge-pair (after (Geisler et al., 2001)). Edge-elements **(A,B)** are considered to be “continuous” if the distances from intersecting lines oriented (dashed lines) are the same. Because of the law of the exterior angle of a triangle, angle θ (the orientation difference) is twice of the angle ϕ (the relative direction).

Natural images were obtained from our own database, as well as those obtained from the McGill Calibrated Color Image Database (Olmos and Kingdom, 2004). These images covered a wide range of scenes, including animals, flowers, foliage, fruits, landscape, shadows, snow, and natural textures (samples of these images are shown in Figure 4A) largely devoid of human structures, though, barring examples of human-made environments such as an unfurnished white-painted room, the statistics of scenes containing human artifacts occupy much the same statistical space as scenes devoid of human artifacts. Images that largely comprised of human-made structures were excluded from this analysis. A sample of 472 pictures was selected based on this criterion. The intensity of images was adjusted according to each image's exposure time so that the calibrated values are linearly related to the absolute luminance of the original scene. As we were only interested in luminance-defined contours, images were then transformed from RGB scale to eight-bit grayscale. To prevent the rectangular aspect of an image from producing unwanted vertical and horizontal edge elements, images were windowed by a circular aperture with a diameter of 480 pixels. Figure 4B shows a processed picture.

#### Edge extraction

Contour analysis was conducted using an automatic edge-detector that faithfully followed the procedures developed by Geisler et al. (2001 see their Appendix A.1). This allowed for comparison with this work and others that have employed this common method of analyzing edge structures in natural images. Edge extraction procedures began with filtering an image using a non-oriented log-Gabor function (with spatial frequency bandwidth of 1.5 octaves, and a peak spatial frequency of 0.1 cycles/pixel):

$$\begin{array}{l} H\left(u,v\right)=\exp\left(-\alpha {\left(\frac{0.5\log\left(u^{2}+v^{2}\right)-\log\left(fc\right)}{Bo}\right)}^{2}\right) \end{array}$$

where *u* and *v* are the horizontal and vertical spatial frequency; *fc* is the peak spatial frequency of the filter, *B0* is the octave bandwidth of the filter, α is a constant of −5.77. Pixels that corresponded to the zero-crossings (within a radius of 216 pixels from the image's center) in the filtered image were considered as edge elements. To illustrate, this procedure was applied to the image shown in Figure 4B and the extracted edge elements are highlighted in red. As shown in this figure, the extracted edge elements corresponded well with the edge of real objects. After the computation of edge structure in natural images, their orientation was computed.

#### Orientation analysis

Edge-element orientation was extracted by filtering the original images using an oriented-log Gabor function (with spatial frequency bandwidth of 1.5 octaves, a peak spatial frequency of 0.1 cycles per pixel, and bandwidth of 40° as per the methods of Geisler et al., 2001) at every 10° of spatial orientation, in both sine and cosine phases:

$$\begin{array}{l} H\left(u,v\right)=\exp(-\alpha {\left(\frac{\log\left(\left|u^{\prime }\right|\right)-\log\left(fc\right)}{Bo}\right)}^{2}\\ \;+i\phi sign\left(u^{\prime }\right)-\log(2){\left(\frac{v^{\prime }}{fc\tan(0.5b_{\theta }}\right)}^{2}) \end{array}$$

where *u*′ = *u* cos θ + *v* sin θ, *v*′ = *v* cos θ + u sin θ, *fc* is the peak orientation, ϕ is the spatial phase, *b*θ is the orientation bandwidth; sign(*u*′) is a function of giving the sign of input variable *u*′. For each filter orientation (0–360° at steps of 10°), sine and cosine filtered images were squared and summed to obtain the orientation-energy for each edge element, which was then normalized by dividing it by the sum of orientation energy across all orientations. A significant edge element corresponded to zero crossing pixels in which the normalized contrast energy across all orientations exceeded 10% of the maximum response, and the edge element orientation was determined by determining the line of best fit along the response distribution.

#### Extraction of contours defined by constant curvature

After extracting the orientation of edge elements from an image using the aforementioned procedure, the geometrical relationships between two edge elements was determined by calculating the distance between the center of the elements (d), the direction of the second element relative to the orientation of the first element (ϕ), and the orientation difference between the elements (θ) (see Figure 4C which is reproduced from Geisler et al., 2001). These parameters served to define the structural criterion for our mapping of different curved contours in natural images and are consistent with the approach adopted by Geisler and colleagues (see Geisler et al., 2001; Geisler and Perry, 2009).

Contours anywhere in the image that possesses the following four distinct criteria (based on the determined geometric relationship between edge elements) were extracted. Note that this process is unguided and elements selected to part of one contour might also be considered with another. This approach is inherent to the methods used in previous contour detection studies. First, curved contours represented fragments consisting of only four equally spaced edge-elements. This restriction was imposed because given the criteria for contour selection contours consisting of many elements are likely to form a circle or wrap around at high curvature angles. Second, contour elements must be “continuous” with each other. An edge-pair in the contour is identified as “continuous” if their center is equidistant from intercepting lines (dotted lines in Figure 4C) oriented in the direction of the two edges. Based on trigonometry, the orientation difference (θ) of a “continuous” edge-pair is twice the direction (ϕ). Third, contour elements have the same inter-element distance with their adjacent elements (with a tolerance of plus or minus one-fifth of the distance). Fourth, they have a constant change of orientation in both sign and magnitude (with a tolerance of ±5° of the orientation change).

Our computational algorithm initially selected an edge element in the image and then searched for continuous edges at a particular distance on both sides of the edge. If a continuous edge was detected this processed continued in the same direction until four continuous edges were revealed. The algorithm then marked the contour in the image and the process was repeated for different contour lengths and curvature angles. Marked contours were then discarded from the search process.

#### Extraction of the orientation of end elements of mapped contours

After identifying the contour using the steps outlined above, for each contour, we determined the orientation of edge elements that are located at the ends of a mapped contour. As mentioned, this parameter revealed the extent to which end elements conformed to the contour angle and our psychophysical data suggests that the orientation of end elements are important in modulating the detection of curved contours. End elements must share the same properties of the mapped contour in that it must be “continuous” (see above) and have the same inter-element distance. Note that our unguided process mapped end elements that are not necessarily located at the end of contours, but can be located in the middle or at any intermediate positions of an actual contour in the image. In our analysis, we only considered continuous end elements with directions that were within −90 to 90° relative to the position of the last element of the curve. This end-element orientation analysis was performed for mapped contours with contour angles of between 0 and 40°.

### Results and discussion

**Figure 5:**
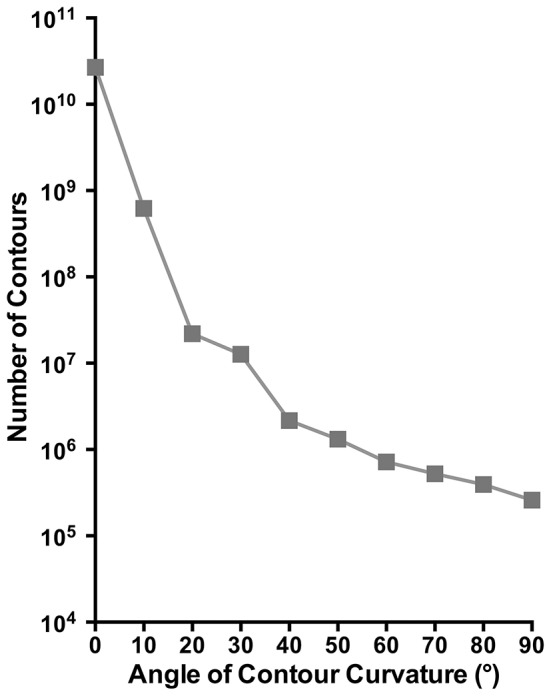
**The total number of curved contours identified in our image analysis plotted as a function curvature angle**.

The total number of mapped curved contours derived from our analysis is plotted in Figure 5 as a function of curvature angle. Evident in this figure is a monotonic decrease in the number of contours as the curvature increases. This finding indicated that contours of different curvatures are not equally prevalent in natural images, but rather contours with small curvature angles occur more frequently. Note that this decrease is less for curvature angles >~40°, which suggests that curves greater than this angle generally occur less frequently in natural images and are equally less prevalent.

**Figure 6:**
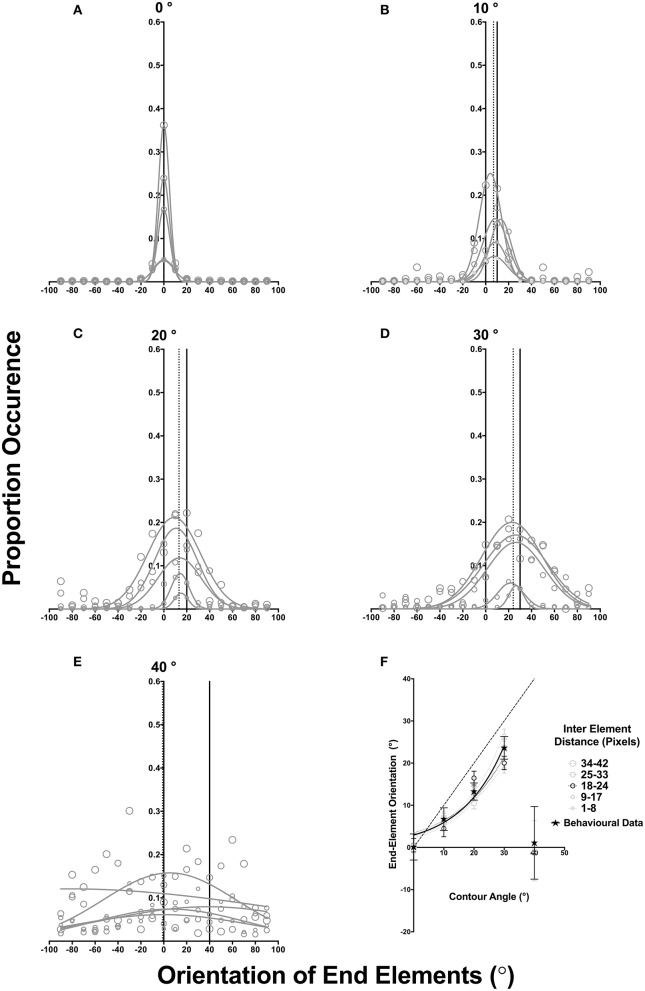
**(A–E)** plots normalized proportion of occurrence for different end-element orientations. Solid and dotted vertical lines indicate the contour angle and the mean peak of the Gaussian fits averaged across all five inter element distance bins. In **(F)** the peak end-element orientation is plotted as a function of the contour angle for our behavioral data (star) and the outcomes of our image analysis; error bars represent SD of the Gaussian fit. Different contour lengths are given by different sized circles (see legend). Dotted lines represent the best exponential fit, while the dashed diagonal line indicates a one to one match between the contour angle and the orientation of end elements.

The proportion of occurrence of end elements is plotted as a function of its orientation in Figures 6A–E, for contours mapped in the natural images with angles of 0, 10, 20, 30, and 40° (different panels). Within each figure, we report data for different contour lengths (size circle symbols) collated within five different bins based on their length [indicated by d which specifies the inter-element distance (bin width of 8 pixels)]. We were unable to report contours with lengths greater than this because contour length is inherently limited by the size of the image (radius of 240 pixels), and longer contours were infrequently mapped in natural images. Accordingly, a clear and reliable relationship between the end-element orientations could not be established for them. To allow for comparison between different contours (as the frequency of occurrence changes with the contour angle see Figure 5), these distributions were normalized to range from 0 to 1 by dividing each value in the distribution by the total number of contours of a particular contour angle. These frequency distributions were fitted with a Gaussian function (as in Experiment 1), which provided an indication of the mean orientation of the distribution, the proportion of occurrence (amplitude at the peak of the distribution), and the orientation range of end-elements corresponding to the standard deviation of the distribution for each of the five contour length bins. Gaussian fits were generally good with R2s > 0.66 for all fitted functions for contour angles of 0, 10, 20 and 30°. However, note that there is a degree of deviation from the best-fit functions particularly at large end-element orientations (in both clockwise and anticlockwise directions) and at large contour angles. These deviations appear to be unsystematic but have reduced the goodness of fit. Note that for a contour angle of 40° Gaussian fits were generally poor (R2s < 0.220) and the end element orientation distribution for all contour lengths were generally very broad.

A number of findings are evident in Figure 6. First, across all five different contour angles (6A-E) the proportion/frequency of occurrence of contours of different lengths (gray symbols) was not equal, but increased with contour length. Linear regression analysis for each contour angle (reported slope and y-intercept values are given below along with their 95% confidence limits) showed that the Gaussian peak value significantly increased with the contour length for contour lengths of 0–30° [0°: slope: 0.0009(±0.001), y-intercept: 0.0005(±0.0286), R2: 0.94, *F*_(1, 3)_ = 53.07, *p* = 0.0053; 10°: slope: 0.005(±0.0001), y-intercept: 0.045(±0.019), R2: 0.91, *F*_(1, 3)_ = 33.85, *p* = 0.0101; 20°: slope: 0.006(±0.0055), y-intercept: 0.03(±0.014), R2: 0.97, *F*_(1, 3)_ = 120.90, *p* = 0.0016: 30°: slope: 0.004(±0.0014), y-intercept: 0.05(±0.021), R2: 0.85, *F*_(1, 3)_ = 17.64, *p* = 0.0246], but not for 40° [slope: 0.002(±0.0013), y-intercept: 0.06(±0.028), R2: 0.43, *F*_(1, 3)_ = 2.28, *p* = 0.228]. However, the same analysis showed no significant change in *SD* for all contour angles (0°: line of best fit: slope: −0.096(±0.060), y-intercept: 8.58(±1.263), R2: 0.45, *F*_(1, 3)_ = 2.49, *p* = 0.213; 10°: slope: −0.036(±0.0414), y-intercept: 10.90(±0.867), R2: 0.21, *F*_(1, 3)_ = 0.77, *p* = 0.444; 20°: slope: 0.422(±0.1441), y-intercept: 9.53(±3.014), R2: 0.71, *F*_(1, 3)_ = 7.25, *p* = 0.0745: 30°: slope: 0.565(±0.1946), y-intercept: 9.73(±4.069), R2: 0.75, *F*_(1, 3)_ = 8.13, *p* = 0.0673; 40°: line of best fit: slope: 2.652(±1.833), y-intercept: 51.06(±38.33), R2: 0.41, *F*_(1, 3)_ = 2.09, *p* = 0.2437);. Note that contours in the two largest length bins (i.e., 25–33, 34–42 pixels) were mapped in natural images with greater frequency than short contours (1–8 pixels). These findings indicate that, contours of constant curvature are likely to be those that span larger spatial distances. The fact that SD did not change suggests that the distribution/tuning of end elements is the same for different contour lengths.

Second, when the results are compared across different contour angles (i.e., across different panels of Figures 6A–E) as the angle of curvature increases, the orientation distribution of end elements broadens. Contours with smaller curvature angles have narrower orientation distributions with the average SD across the five length bins equal to 6.525°For contour curvature of 0°, but this increased proportionally with the contour curvature angle. This data trend was consistently observed across all contour length bins (different gray symbols) with the average SD for contour angles of 10, 20, 30, and 40° approximately 12.24, 17.12, 23.92, and 75.2° respectively. Linear regression analysis examining the relationship between contour angle and SD showed a significant change in the slope of the line of best fit (0°: slope: 0.4479(±0.063), y-intercept: 4.429(±1.544), R2: 0.94, *F*_(1, 3)_ = 50.46, *p* = 0.0057). Thus, with increasing angle of curvature, the range of mapped end-elements broadens.

Third, as the angle of contour curvature increased, there was a systematic deviation in the mean of the frequency distribution in the curvature direction, and this was consistently observed for different contour length bins. For 0, 10, 20, and 30°, the distribution mean averaged across all five contour length bins (dotted vertical line) corresponded to end-element orientations of approximately 0 ° (SEM: 0.0193°), 7.02° (SEM: 1.034°), 13.21° (SEM: 0.377°), and 24.05° (SEM: 0.9051°). Thus, in natural images, end elements that are oriented clockwise to the angle of curvature occur more frequently. Additionally, these average offsets (averaged across the five different length bins: dotted vertical line) do not entirely agree with the angle of curvature (solid vertical line), mirroring a similar effect with our behavioral data (see Figure 6F). Note that these results are not observed for a 40°Contour, regardless of the contour length; the average peak is −0.7992° (SEM: 7.81°), but the average SD distribution is flat and broadly tuned to the end-element orientation (see Figure 6E).

In Figure 6F we report the peak frequency orientation of the fitted distribution for the five different length bins as a function of the contour angle. The average peak frequency orientation for the different length bins are shown individually (different size circles, see figure legend) for contour angles of 0–30°, but only the average across all length bins is shown for 40°. Error bars represent 1 SD of the fit value. As shown in this figure, generally, end elements are oriented in the curvature direction for different lengths, suggesting that this contour property persists over a range of contour lengths. These findings are consistent with previous studies that have shown that the statistics of natural images are largely scale invariant (e.g., Field, 1993; Ruderman and Bialek, 1994), and those by Geisler et al. (2001) and Sigman et al. (2001) who have shown that the pairwise relationship between edge elements might also be scale invariant and persist over a range of separations. This monotonic increase for different contour sizes is well modeled (average R2 > 0.934) by an exponential growth equation of the form:

$$\begin{array}{l} Y=S\;{\text{exp}}^{\left(K-X\right)} \end{array}$$

where *S* is the starting value and *K* is constant. Best fit values (with estimated 95% confidence intervals) for each length bins were: 1–8 pixels: *S*: 3.017 (±1.279), *K*: 0.074(±0.0152); 9–17 pixels: *S*: 3.559(±1.723), *K*: 0.057(±0.0181); 18–24 pixels: *S*: 3.490(±2.294), *K*: 0.060(±0.0243); 25–33 pixels: *S*: 2.308(±0.846), *K*: 0.074(±0.013); 34–42 pixels: *S*: 2.623(±0.951), *K*: 0.070(±0.013). However, note again that the bias in curvature direction is always less than the curvature angle (dotted diagonal line).

## General discussion

Our study reports a number of findings. In Experiment 1 we showed that sensitivity to curved contours decreases as a function of curvature angle (replicating the findings of Pettet, 1999) and is dependent on the orientation of end contour elements. The latter finding demonstrating that the visual system is sensitive to the contour curvature angle and disruption to its continuity attenuates detection. In Experiment 2, we established that this effect is dependent on the separation of end elements and is selective for contours that are C-shaped and not S-shaped. Finally, we undertook a focused image analysis, which revealed that curved contours in natural images demonstrate good continuity in curvature structure with end elements likely to follow the contour angle.

Note that a direct comparison is not possible between our psychophysical data and our image analysis given the very different stimulus conditions and assumptions in the two studies. Particularly, in Experiment 1 we only examined detection of curved contours of a fixed in length and end elements were actually the last element of the contour. While in our image analysis, mapped contours were of various lengths, and end elements could be intermediate end elements of an actual contour in the image. Nevertheless, there is broad agreement between our behavioral data and the outcomes of the image analysis. Particularly, it can be observed that the visual system is sensitive to curved contour structure, and this sensitivity might conform to the regular structure of curved contours in natural images. For comparison, we have plotted our behavioral data (observer-average Gaussian mean represented by star symbols) along with the outcomes of our image analysis in Figure 6F. Additionally, we have highlighted data for contours with lengths between 18 and 24 pixels (black circles), as the length of the contour used in our behavioral study was within this length bin. Again there is agreement between our psychophysical data and this contour length condition. However, we emphasize that further investigation is necessary to provide a more comprehensive assessment of the dependency of visual behavior on scene statistics. For example, it will be critical to examine the extent of the detection bias for curved contours with different lengths and correlating them with their occurrence frequency in natural images.

Our finding suggests that the visual system specifically codes contour curvature. This observation agrees with the work of Gheorghiu and Kingdom (2007a,b, 2008) who employed shape frequency and shape-amplitude after-effects to probe the sensitivity of the visual system to curvature structure. For example, Gheorghiu and Kingdom (2007a) noted that adaptation to a sinusoidal shaped contour resulted in shifts in the perceived shape-frequency and shape-amplitude of a test sinusoidal grating. They observed that these illusions are a result of adaptation to local contour curvature, as the after-effect reached maximum when only half a cycle of the test stimulus was present. Additionally, using the shape-frequency and shape-amplitude after-effect as a probe for curved contour processing, Gheorghiu and Kingdom (2008) demonstrated that curvature-coding mechanisms were dependent on the local orientation of elements, curvature polarity (i.e., sign of curvature) and shape phase.

In Experiments 1 and 2 we noted a detection advantage for C-shaped contours when end elements were cocircular, relative to when they were collinear. However, an opposite result was observed with S-shaped contours; here collinear end-elements lead to superior detection relative to when they were cocircular. A possible explanation for the difference in finding might reflect the efficiency with which local contour elements are grouped. As noted by Field et al. (1993), the basis upon which contour elements are associated reflects well-known Gestalt grouping principles such as continuity, smoothness, and proximity. It is possible that the visual system relies on the principles of good continuity and smoothness such that the grouping of elements further facilitates the grouping of additional elements. Thus, for C-Shaped contours, because the central four elements were continuously and smoothly curved, this might promote the grouping of end-elements oriented clockwise to the angle of curvature. However, for S-shaped contours, the bias toward grouping is weaker because of the sign change in curvature in the middle of the contour. Under these circumstances, their detection is biased for collinear end-element arrangements.

Another possible contributing grouping factor involved in the processing of curved contours is the principle of closure, which has been shown to facilitate contour detection (see Kovacs and Julesz, 1993). While in the present study we employed “open” contour fragments that were not closed, the curvature of such contours (particularly at large contour angles and for end-element orientations in the direction of curvature) might signal a “closed” unit, because of a tendency by the visual system to perceive a whole stimulus from incomplete parts. This, in turn, might facilitate the detection of curved contours. The present study did not compare “open vs. closed” curved contours, nor did our experimental allowed for their direct comparison. We acknowledge the possible role of the principle of “closure,” and more focused studies will need to quantify its selective contribution the detection of curved contours. For example, the experimental procedures of Gheorghiu and Kingdom (2007a) could be used to determine the dependency and extent of the shape after-effects (in an open and or closed test contours) from selective adaptation to open and closed curved contours shapes.

Yen and Finkel (1998) presented a computational model in which contour detection in the context of curvature can be accounted for. This model proposes that in the formation of contours, edge elements will be associated with elements others, given that their relative orientations and position conform to the principles of the association field model of Field et al. (1993), however, no association is made between elements in which the angular difference is too large (but falls along the angle of curvature). However, according to Yen and Finkel, if information about constant curvature were retained in lateral connections this bias would “steer” the relative weighting of the connection field in the angle of curvature, and in this way association of elements that lie along the angle of curvature (but would not be associated, if adhering strictly to a collinearity principle) are formed. Our behavioral data agrees with this model. In light of the findings of the present study (and those by Geisler et al., 2001; Sigman et al., 2001), it could be argued that the coding of curved contour structure at early stages of visual processing (as proposed by Yen and Finkel, 1998) is driven by image statistics. Particularly this computational approach ensures efficiency of processing because the visual system is selective for contours that occur with greater frequency in the natural world and provide an effective means of extended grouping before object recognition, which overcomes local luminance variations which would otherwise affect the detection of contours (see Elder and Goldberg, 2002).

It has been reported that the visual system is sensitive to regular deformations of a circular shapes as revealed by examining the detection of radial frequency patterns (see Wilson and Wilkinson, 1998) or Glass patterns (see Or et al., 2010; Khuu et al., 2011). Such radial frequency patterns are defined by a circular shape, but with regular deformations conforming to a cosine modulation. Previous studies have provided evidence for neural mechanisms capable of detecting the shape conveyed in radial frequency patterns (e.g., Poirier and Wilson, 2006) A possibility is that the contours investigated in the present study are detected by such mechanisms and our findings represent sensitivity to deformation along the extent of the curvature edge. However, were such detectors responsible for the detection of curved contours used in the present study, it would be expected those end elements that deviated greatest from the curved contour angle (producing greater deformation regardless of whether it is clockwise or anticlockwise in direction) would lead to greater detection. However, this was not observed, but rather only a slight orientation clockwise to the curvature direction lead to best detection. It is possible that our psychophysical data assesses functioning before the integration of contour fragments for the detection of circular shapes (like radial frequency patterns), and reflects local operations in primary visual cortex that initially associate local elements into contour fragments. As concluded by the present study and others, this process might be guided by the statistics of natural images.

While we have attempted to account for our findings in terms of the functioning of local edge detectors, our data cannot rule out the influence or possibility that global detectors are involved in the processing of contour curvature. Indeed, recent fMRI experiments have revealed feedback inputs to lower cortical areas from higher regions of the visual field (such as the LOC) are involved in the perception of contours (see Tanskanen et al., 2008; Mijovic et al., 2013). Previously we have accounted for the results of the present study by suggesting the involvement of grouping principles such as continuity, smoothness, and closure. It is possible that neural functioning and feedback processes might reflect neural implementation of such grouping operations. However, future neural imaging studies will be useful in clarifying the involvement of global detectors in the perception of curved contours.

It is important to note that validity of image analysis lies on the assumptions that the techniques employed provide a good reflection of the operations employed by the visual system to extract edges from the visual image. To date, and a major issue is that different types of edge operators (with their own inherent theoretical assumptions) have been proposed to account for the functioning of the visual system. While the techniques used in the present studies adhere to those used by Geisler et al. (2001), they are not without their limitations. For example, Elder and Goldberg (2002) noted that in Geisler et al.'s study that the relationship between edge-pairs was made regardless of their ordinal distance in the chain. The consequence of this operation is that the statistics relating to neighboring elements is diluted by the weak statistics relating to distant elements. This certainly might affect the mapping of longer curved contours as its curvature structure becomes coarsely sampled (as our approach was limited to four elements). Because our analysis procedures were mainly based on Geisler et al. (2001) study, this limitation is inherited. However, in a recent study Geisler and Perry (2009) have validated and established the effectiveness of their contour analysis method in synthetic images in which their content are known. Additionally, as our analysis of contours remained unguided it is possible that the search algorithm might result in spurious identification of a contour. This might occur when line elements from separate contours overlap or intervene, and provided that criteria adopted by the present study are met, will be identified as the same contour. However, the similarity between our psychophysical and natural image analysis (see Figure 6F), would suggest this issue might have only a minimal impact on their relationship. Future studies might wish to adopt a more guided approach (see Geisler and Perry, 2009) in which observers first identify and label contours in the images.

## Ethics statement

This study was approved by the Human Research Ethics Advisory committee at the University of New South Wales and the research ethics committee at the University of Hong Kong Human participants gave their informed consent and signed consent forms to indicate that participation in the study.

## Author contributions

SK, JC and AH. contributed equally in conducting the study and writing the manuscript.

### Conflict of interest statement

The authors declare that the research was conducted in the absence of any commercial or financial relationships that could be construed as a potential conflict of interest.
